# Using Biosensors to Study Organoids, Spheroids and Organs-on-a-Chip: A Mechanobiology Perspective

**DOI:** 10.3390/bios13100905

**Published:** 2023-09-24

**Authors:** Muhammad Sulaiman Yousafzai, John A. Hammer

**Affiliations:** Cell and Developmental Biology Center, National Heart, Lung and Blood Institute, National Institutes of Health, Bethesda, MD 20892, USA

**Keywords:** biosensors, organoids, spheroids, extracellular matrix (ECM), mechanobiology, organ-on-a-chip (OoC)

## Abstract

The increasing popularity of 3D cell culture models is being driven by the demand for more in vivo-like conditions with which to study the biochemistry and biomechanics of numerous biological processes in health and disease. Spheroids and organoids are 3D culture platforms that self-assemble and regenerate from stem cells, tissue progenitor cells or cell lines, and that show great potential for studying tissue development and regeneration. Organ-on-a-chip approaches can be used to achieve spatiotemporal control over the biochemical and biomechanical signals that promote tissue growth and differentiation. These 3D model systems can be engineered to serve as disease models and used for drug screens. While culture methods have been developed to support these 3D structures, challenges remain to completely recapitulate the cell–cell and cell–matrix biomechanical interactions occurring in vivo. Understanding how forces influence the functions of cells in these 3D systems will require precise tools to measure such forces, as well as a better understanding of the mechanobiology of cell–cell and cell–matrix interactions. Biosensors will prove powerful for measuring forces in both of these contexts, thereby leading to a better understanding of how mechanical forces influence biological systems at the cellular and tissue levels. Here, we discussed how biosensors and mechanobiological research can be coupled to develop accurate, physiologically relevant 3D tissue models to study tissue development, function, malfunction in disease, and avenues for disease intervention.

## 1. Introduction

**Figure 1:**
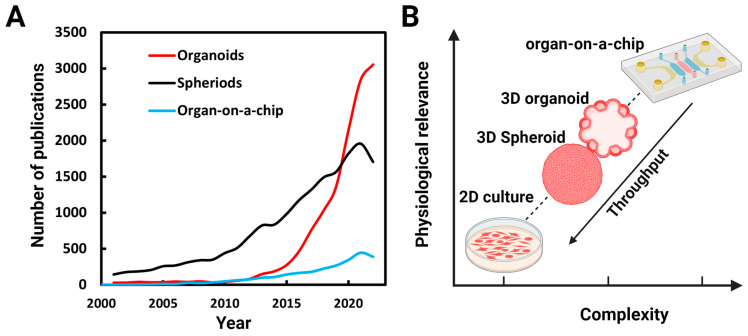
Numbers of publications on organoids, spheroids, and organs-on-a-chip: (**A**) Number of publications per year found identified by a PubMed search using the terms organoids, spheroids (spheroids and cell aggregates), and organ-on-a-chip between 2001 and 2022. (**B**) As we get closer to in vivo conditions, the complexities of the systems increases and throughput decreases.

The culture of cells in 2D has powered tremendous advances in the biological sciences and in the biotech industry [1]. Two-dimensional cultures are easy to make and manipulate, relatively inexpensive, and for immortal cells last indefinitely. That said, major differences exist between cells grown in 2D and in 3D at both the single cell and collective cell levels. For example, invasive cells use different modes of migration in 3D culture than in 2D culture [2]. Similarly, epithelial cells undergo geometrical and structural transformations in 3D culture that do not occur in 2D cell monolayers [3]. As a case in point, the modeling of brain development is possible using 3D organoids but is not possible using 2D cultures [4]. One major disadvantage of 2D cultures is that they lack many of the cell–cell and cell–matrix interactions seen in tissues. Such interactions likely influence responses to pharmacological perturbations. The fact that cells in 2D are usually homogenous, i.e., not in contact with other cells types as in tissues and many types of organoids, may also influence responses to pharmacological perturbations [5]. On the other hand, 3D cultures provide in vivo-like cell–cell and cell–matrix interactions, and the associated signaling pathways responsible for affecting cell phenotypes. However, 3D cultures are more costly to establish and maintain than 2D cultures. They also rely largely on imaging and manipulation modalities designed for 2D, and so, there are some methodological challenges to overcome [6]. These prominent differences can lead to many differences in biological functionalities, and hence, our fundamental understanding of in vivo processes (Figure 1).

**Figure 2:**
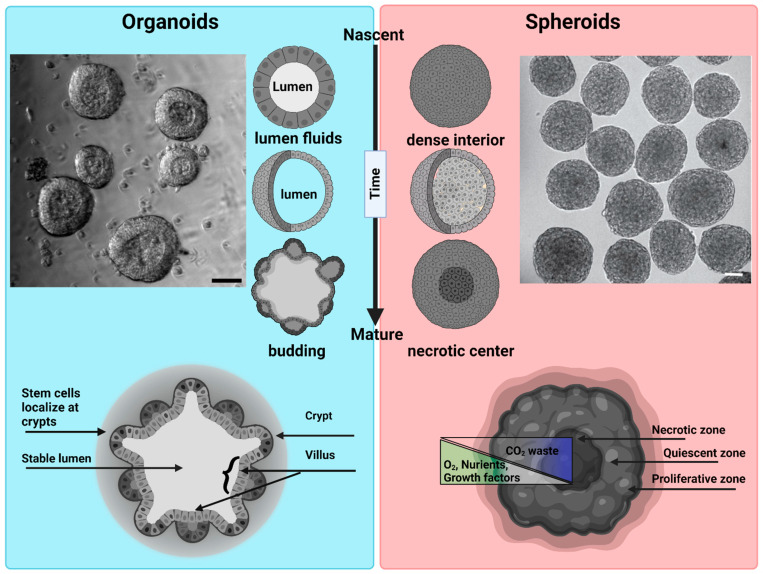
Morphological differences between organoids and spheroids: Organoids: a representative bright field image of intestinal organoids grown in Matrigel from an intestinal crypt. Nascent organoids have a spherical morphology, are composed of a monolayer of a columnar stem cells and transient amplifying cells, and contain a lumen filled with fluid and extruded cells. As they mature, organoids grow in size and develop a crypt and villus morphology just like intestinal tissue in vivo. The stem cells are localized within the crypts, while the nutrient-absorbing enterocytes form the villus. Intestinal organoids can be used to study mechanisms of barrier function, lumenogenesis, cell division, and cell extrusion. Spheroids: a representative DIC image of spheroids formed by Sarcoma S-180 cells formed via a spinning method within a non-adherent platform. Adopted with permission from [21]. Nascent spheroids are initially roughly spherical, and then merge together to form big spheroids. They are dense structures where cells are connected initially through cell–cell-adhesion. As they mature, they produce necrotic centers. Spheroids are very useful for measuring tumor growth dynamics, mechanical and immuno-resistant barriers, drug responses, and solid stresses. Scale bar is 50 µm.

Different terminologies are used by the research community for 3D cellular structures. These include organoids, enteroids, spheroids, cell-aggregates, and organotypic cultures. Importantly, some of these structures differ as regards their structure and function, as well as their phenotypic and genotypic complexity [7]. That said, we can broadly categorize 3D structures as organoids and spheroids, as both are 3D cell culture systems that mimic to varying degrees the structure and function of an in vivo tissue. An organoid is a 3D cell culture system derived from stem cells or tissue progenitor cells. They have the ability to self-organize, regenerate, and recapitulate to varying degrees a tissue-specific phenotype [8]. Organoids are broadly used to study organogenesis of homeostatic and diseased organ tissues [9]. A spheroid is a 3D culture derived from a cell line that under the right conditions will aggregate to form a 3D spherical structure [10]. While spheroids can be grown with or without a matrix, they are most commonly created using nonadherent conditions to achieve high yield and rapid, synchronous formation. Spheroids are commonly used as simple tumor models [11,12,13] (Figure 2).

Cells in vivo are constantly probing their mechanical environment through integrin-based adhesions to the extracellular matrix (ECM), cadherin-based adhesions to surrounding cells, and mechanosensitive ion channels. They then integrate all of these signals to trigger an appropriate response [14]. As a result, tissues exhibit characteristic mechanical features that ensure proper tissue functionality. For example, brain tissues are soft with elastic moduli of ~100 Pa, while bones are very stiff with a modulus of over 100 kPa [15]. While the mechanical features of tissues are mostly invariant under homeostatic conditions, that can change in pathological conditions. For example, tumors are typically stiffer than the surrounding healthy tissues [16]. A good example of this is breast cancer, where normal breast tissue is in the range ~500 Pa and increases to more than 1000 Pa in breast tumors. Importantly, 3D spheroids and organoid models can be exploited to understand the connection between the mechanical signature of a tissue and its functionality in vivo. Generally, Matrigel and Collagen type-I are the most common matrices used for generating 3D cultures [17]. Matrigel is mostly used for growing organoids while collagen I is mostly used in tumor invasion studies as it better mimics the tumor microenvironment [7,18]. Of note, there are batch-to-batch variabilities in the composition of these matrices and, hence, variabilities in their mechanical properties. Moreover, because they are not amenable to chemical and mechanical alterations, it is hard to fine-tune matrix mechanics to promote a specific cell behavior. Given this, there is a clear need to design mechanically controllable matrices with precise chemical compositions and mechanical properties [19,20].

To understand cell behavior in 3D environments, and to develop mechanically tunable matrices for such environments, one must possess tools capable of accurately measuring forces in 3D. Our current understanding of mechanosensing and mechanotransduction relies mainly on mechanical and optical biosensors that have been optimized for use in 2D contexts. Adapting these mechanical and optical biosensors to organoids and spheroids holds great potential for advancing our understanding of cellular behaviors, disease processes, and drug responses in a more physiological context.

Organoid growth involves complex shape transformations driven by mechanical forces generated in large part by actomyosin-dependent contractions. These forces are constantly adjusted by cell–cell and cell–matrix crosstalk to establish the correct morphological structure for the given environmental condition. In organoid cultures, the structure emerges stochastically as there is no control over stem cell differentiation patterns or mechanical forces. To remedy this, organ-on-a-chip (OoC) platforms can be used to precisely control structure by defining the position of stem cells and by allowing the application forces with increased spatiotemporal resolution.

Due to their potential for both basic and translational research, many research groups have begun using 3D culture platforms. A survey of PubMed (www.ncbi.nlm.nih.gov, accessed on 27 June 2023) (Figure 1A) over the last two decades shows a corresponding surge in research publications. Systems complexities increase as we get closer to physiological conditions in vitro and throughput decreases (Figure 1B).

This review focused on experimental approaches that can be used to harness organoids and spheroids as models for mechanobiology and bioengineering studies. In particular, we discussed the importance of efforts to recapitulate in vivo like characteristics when using 3D culture systems, as this is essential to answer basic biological questions in tissue differentiation, structure, function, homeostasis, and disease progression. We also reviewed preparation methods and crucial features of the biochemical and mechanical properties of matrices. Finally, we reviewed in depth the optical and mechanical biosensors that are available to study the mechanobiology of organoids and spheroids, and we discussed how organs-on-a-chip might revolutionize the study of diseases and personalized medicine.

## 2. Optical Sensors in Mechanobiology

Optical sensors play a central role in the study of mechanobiology by enabling the accurate detection and measurement of mechanical forces and their effects on biological systems. The history of biosensors dates back several decades to when the term “biosensor” was first introduced by Clark and Lyons in 1962 to describe a device that used an enzyme electrode to measure glucose levels [22]. An enormous advance in the field of biosensors was the discovery of Green Fluorescent Protein (GFP) [23]. By allowing one to observe the dynamic activities of proteins within living cells in real time, GFP profoundly reshaped the landscapes of imaging, cell biology, and optical biosensing. Mechanobiology explores how mechanical forces influence cellular processes, tissue development, and overall physiological functions [24]. Unlike chemical sensors, which can be studied from the analyte concentrations, force cannot be directly measured; it can only be inferred from material deformations [25]. Harris, Wild, and Stopak published a seminal paper in 1980 in which they measured cell-generated forces by observing wrinkles in a silicone substratum, thereby laying the groundwork for traction force microscopy (TFM) [26]. In 1995, Guilford and colleagues employed micro rheology to measure internal cellular forces by utilizing magnetic beads that were internalized by macrophages [27]. The integration of Förster Resonance Energy Transfer (FRET) in cells [28], the development of force spectroscopy techniques (optical tweezers, magnetic tweezers, atomic force microscopy), and super-resolution microscopy (SIM, STED, STORM, PALM) have all changed the course of mechanobiology.

While the field of mechanobiology has experienced enormous growth over the last few decades, there is still ongoing debate regarding the time and length scales over which cell mechanics actively contribute to biological processes [29]. Cells have the ability to sense (outside-in), generate (inside-out), and integrate mechanical forces over a range of time and length scales [30]. The cell’s plasma membrane is the primary site of force transmission. Moreover, it is decorated with mechanosensitive channels (i.e., Piezo1, Piezo2) and transmembrane receptors (e.g., integrins) that link the cell’s interior to the outside word. When subjected to mechanical stimuli, mechanosensitive channels respond within milliseconds. Conversely, integrin-mediated adhesion interactions take tens of seconds, and the cytoskeletal alterations that occur downstream of force transmission through integrins occurs over the span of many minutes. Likewise, cells also receive mechanical forces from neighboring cells in the tissue [31]. Cell integrate these mechanical forces to organize and control global responses like cell migration, proliferation, cell extrusion, and structural/morphological changes [32]. How cells integrate these forces to create global responses is only partially understood. Efforts to quantify mechanotransduction at different time and length scales, and to correlate these measurements with physiological responses, will provide major insights.

Prominent mechanobiological techniques that utilize optical sensors include TFM, optical tweezers, and FRET-based tension sensors. While it is not possible to fully understand mechanotransduction using just one technique, results obtained using multiple techniques can be combined to obtain deeper understanding of any particular event.

**TFM** has played a major role in the growth of the field of mechanobiology. This technique is used to quantify the forces that cells exert on their surrounding environment [33]. It is most commonly used to study cell–substrate interactions, where cells adhered to a deformable substrate like a hydrogel can generate mechanical forces. These forces are quantified by imaging fluorescent microbeads embedded within the substrate. As the cell applies force to the substrate, the beads in the substrate are displaced. By tracking bead displacements using particle image velocimetry (PIV), the distribution and magnitude of the forces exerted by the cells on the substrate can be calculated. While TFM is typically used to measure forces produced by single cells [34], and even forces generated by individual focal adhesions [35], it can also be used to measure forces produced by collections of cells (e.g., 2D organoids) [36]. Studies employing TFM have provided major insights into the mechanobiology of cell migration [37], cell division [38], wound healing [39], and jamming- unjamming transitions [40]. That said, the implementation of TFM in 3D systems will be challenging as the technique relies on displacements fields of ECMs with known mechanical properties, which is not the case for 3D models like organoids and spheroids. TFM in 3D is further discussed in Section 6.

**Optical tweezers** use focused laser beams to trap and manipulate microscopic objects such as cells and beads [41]. By harnessing radiation pressure and momentum, optical tweezers create a localized region of higher intensity where particles experience a trapping force. This force can be precisely controlled to manipulate and measure the displacements of individual cells, particles, or even single molecules in the range of 1 nm to 1 mm [42]. In the context of mechanobiology, optical tweezers are used to apply and measure forces at the pico-newton (pN) level. This powerful technique has provided major insights into the biomechanical properties of single cells and the role of mechanical forces in various biological processes. The high sensitivity of advanced optical tweezers has also provided a powerful tool for investigating the biophysical properties of single molecules like motor proteins [42,43]. The application of optical tweezers to 3D cell culture models is so far limited, although it has been used successfully to measure the viscoelasticity of cells within organoids [44].

**FRET** has evolved from a theoretical concept [45] to a widely used experimental technique with diverse applications in cellular and molecular research [46]. Indeed, FRET sensors have revolutionized our ability to interrogate mechanical forces and dynamics within living cells [47] by providing insights into how cells respond to mechanical cues, how forces are transmitted across cellular structures, and how mechanical processes contribute to cellular behaviors. Moreover, the constant improvements being made to FRET-based technologies should ensure even deeper insights into the mechanics of tissues, cells and molecules. FRET is a fundamental molecular phenomenon that involves the transfer of energy from an excited donor fluorophore to an acceptor fluorophore when they are in close proximity [48]. Because energy transfer is extremely sensitive to the distance between the donor and acceptor molecules, FRET provides a very powerful tool to study interactions between different molecules, as well as conformational changes within individual molecules [49]. These genetically encoded tension sensors can be used to measure mechanical forces within living cells by revealing mechanically responsive domains within individual proteins tagged appropriately with both the donor and acceptor probes as changes in fluorescence intensity, spectral shift, or other measurable signals [48]. These sensors have been used extensively to visualize interactions and quantify mechanical forces exerted on specific cellular components such as cadherin-based cell–cell adhesions, integrin-based cell–substrate adhesions [50], membrane–membrane interactions [51,52], and cytoskeletal structure interactions [53]. Although FRET has proven very valuable for investigating molecular interactions, its utilization in the context of 3D mechanobiology presents challenges as regards spatial resolution, penetration depth for optical imaging, quantification accuracy, background noise, microenvironment variations, cellular diversity, and other technical complexities.

**Figure 3:**
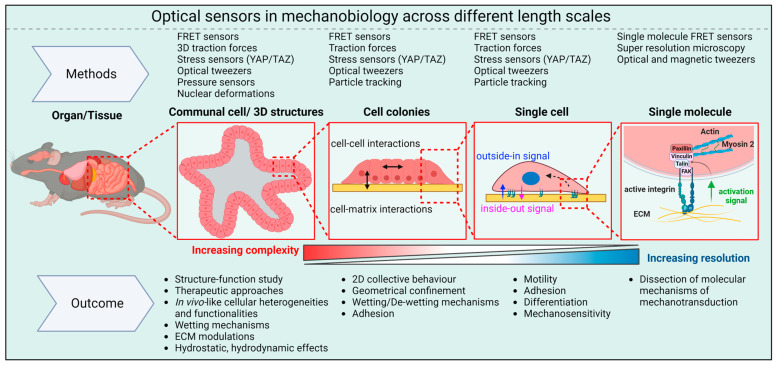
Optical sensors in mechanobiology: Optical sensors can be used across a wide range of length scales. At the molecular level, they provide insights into the pN forces involved in mechanotransduction inside cells. On larger scales, these intracellular forces combine with forces between cells and between cells and the ECM to shape the overall 3D structure of tissues and organs.

While the advanced methods discussed above have provided many insights into molecular aspects of mechanotransduction, interpreting these results in the context of tissues (e.g., tissue function, differentiation, and remodeling) poses significant challenges. Indeed, the development of techniques that allow biosensors to be used in 3D cellular structures like organoids and spheroids will be particularly important. For example, measuring cellular-scale forces within organoids, spheroids, and tissues remains very challenging because 3D ECMs are quite complex. The optical biosensors currently employed to study the mechanobiology of organoids and spheroids are summarized in Figure 3, and are described in detail in Section 6.

**YAP/TAZ**: YAP-TAZ (Yes-associated protein (YAP) and WW domain-containing transcription regulator protein 1 WWTR1, also known as TAZ) are transcriptional coactivators that play a pivotal role in several fundamental cellular processes, including cell proliferation and tissue development. When cells are subjected to mechanical stimuli or experience changes in their mechanical environment, YAP translocate into the cell’s nucleus where it acts together with various transcription factors to regulate gene expression. Because YAP/TAZ responds to mechanical cues, fluorescent versions can be used as a mechanosensors to define the cell’s mechanical state [54]. YAP has been shown to regulate intestinal stem cells [55] (YAP activation is higher in the stem-cell rich crypt than in the villus [56]), and transient YAP activation is necessary for symmetry breaking in organoids [57].

**Nuclear deformation**: Forces impinging on the nucleus can alter nuclear shape, chromatin structure, and, ultimately, the cell’s transcriptional profile. The dynamic visualization of cell nuclei using GFP or RFP tagged with a nuclear localization sequence (NLS), GFP-tagged Histone H2B, or even the DNA dye DAPI have the potential to serve as mechanical sensors in 3D cell models. Because nuclear volume scales with cell volume [58], the orientation, size, and shape of the nucleus can be employed as an indicator of the cell’s mechanical surroundings [21,59].

**Pressure sensors:** Cells present within 3D structures are exposed to a wide variety of forces that include hydrostatic pressure, compression, tension, and shear stress. These forces are created by cell compaction, by fluid filled lumens, and by changes in the microenvironment. Our understanding of how these stresses and pressures alter cellular behavior and influence 3D morphology is held back by a lack of proper tools to accurately measure such forces within tissues. Of note, cell-sized mechanical sensors have used been to detect internal stresses within spheroids [60]. These sensors are made of fluorescent polyacrylamide [61] or oil droplets [62], both with known mechanical properties.

In addition to the optical sensors discussed above, Brillouin microscopy and Elastic Resonator Interference Stress Microscopy (ERISM) are increasingly being used to measure the mechanical properties of tissues.

**Brillouin microscopy** is an advanced imaging technique that utilizes the phenomenon of Brillouin scattering to characterize the mechanical properties of cells and tissues [63]. By measuring the frequency shift of scattered light due to acoustic waves propagating through the sample, this method can be used to map variations in material stiffness and elasticity at the microscopic level [64]. Brillouin microscopy offers a non-invasive and non-destructive means to evaluate intracellular stiffness at submicron resolution [65].

**ERISM** is an innovative microscopy technique that employs a micro-resonator (typically made of an elastic material) as a probe to measure the mechanical properties of cells [66]. The interaction between the resonator and the cell creates interference patterns that reveal variations in stress and mechanical properties.

Importantly, comprehensive knowledge regarding the composition and mechanical properties of the matrices that support 3D structures is essential for the proper analysis and interpretation of biosensor data. In this regard, the development of 3D matrices with tunable mechanical properties would greatly accelerate efforts to characterize the mechanobiology of organoids and spheroids. Such an effort will require accurate measurements of the mechanical properties of 3D cell cultures and determining how these properties can be harnessed to advance biosensor development.

## 3. The Importance of Matrix Selection for Organoids Growth

The most commonly used matrix for culturing organoids is Engelbreth–Holm–Swarm (EHS) matrix, which is a reconstituted basement membrane harvested from mouse sarcoma [67], and which is known by the trade names Matrigel, Geltrex, and Cultrex BME [17]. EHS matrix contains various ECM components including laminin, collagen type IV, entactin/nidogen, growth factors, and proteoglycans secreted by EHS cells [68]. The density of binding sites for cellular adhesion molecules in this matrix is sufficient to support robust organoid growth and differentiation. Moreover, Matrigel can be degraded and remodeled by cellular proteases and cell-induced mechanical stresses. Other naturally derived ECMs like collagen I, gelatin, fibrin, and decellularized ECM have also been used to grow organoids and spheroids. Synthetic hydrogels, including polyethylene glycol (PEG), nanocellulose, and alginate have been used as well [69]. Both natural and synthetic ECMs have specific chemical compositions, densities of adhesive ligands, pore sizes, and rigidities (Figure 3A) [70]. It is important to note that Matrigel exhibits some batch-to-batch variability, which can complicate data interpretation, especially in the context of translational studies like drug screens. Additionally, it cannot be easily tailored to meet the diverse requirements of unique organoid niches. As a result, efforts are now being made to design synthetic ECMs that are both well-defined and amenable to precise manipulation of their chemical and mechanical properties.

Cells bind to ECM through integrin receptors, which then recruit focal adhesion proteins and cytoskeletal elements to establish stable adhesions that are capable of transmitting and transducing forces. Integrins transduce signals bidirectionally, i.e., both inside-out and outside-in [71]. In mammalian cells, integrins can be broadly categorized into laminin binding integrins (α1β1, α2β1, α3β1, α6β1, α7β1, and α6β4), collagen binding integrins (α1β1, α2β1, α3β1, α10β1, and α11β1), and RGD-binding integrins (α5β1, α_V_β1, α_V_β3, α_V_β5, α_V_β6, α_V_β8, and αIIbβ3) [72]. Establishing the appropriate integrin–ligand adhesion pair is crucial for tissue morphogenesis. For example, Shuoran Li et al. showed that endothelial cells must engage α_v_β_3_ integrins to promote vascular sprouting, and α3/α5β1 integrin to promote vasculature maturation [73]. Similarly, Stanton et al. showed using mesenchymal stem cells plated on the four major ECM proteins (fibronectin, collagen I, collagen IV, and laminin) that YAP translocation is strongly influenced by the ECM type [74].

Collagen I is one of the most abundant proteins in the human body, with prominent expression in bone, skin, and connective tissues. Consistently, collagen I is used extensively in cancer research to study collective cell migration and metastatic cell invasion. Collagen IV, on the other hand, is the main component of Matrigel and plays a major role in organoid growth. Collagen I is composed of two types of α-chains (α1 and α2), whereas collagen IV consists of six α-chains (α1–6). This difference underlies their classification as fibril-forming and network-forming architectures, respectively. Importantly, integrins demonstrate distinct specificity for collagen. For example, integrin α1β1 exhibits a higher affinity for collagen IV, while integrin α2β1 binds more strongly to collagen I [75,76].

Another important parameter that can influence the growth and function of organoids and spheroids is *ligand density*. In a study by Beaune et al. [77], ~100 μm diameter murine sarcoma cell aggregates were allowed to spread on polyacrylamide gels with rigidities ranging from 2 kPa to 40 kPa, and containing 0.1 mg/mL fibronectin. While aggregates spreading on the stiffer substrate formed an isotropic monolayer resembling the wetting of a liquid droplet, aggregates spreading on the soft substrates (below 16 kPa) exhibited anisotropic spreading and de-wetting. These differences resulted in differences in cell motility mechanisms. In a subsequent study by Yousafzai et al. [78], aggregates were shown to spread completely on polyacrylamide gels with stiffnesses ranging from 0.7 kPa to 40 kPa when a fibronectin concentration of 1 mg/mL was used. Together, these results reveal an ECM density-dependent response of cellular aggregates to stiffness. In another example, Kenneth Yamada and colleagues [79] showed that increasing the content of laminin in Matrigel, which is normally 60% laminin and 30% collagen IV, reduced the budding of salivary gland organoids. Interestingly, budding was also inhibited when matrix metalloproteinases were inhibited. These findings highlight the significance of matrix composition and ECM remodeling in budding morphogenesis.

Robust stem cell differentiation is essential for proper tissue morphogenesis both in vivo and in vitro. Lutolf, Clevers, and colleagues [80] investigated the effects on organoid growth of different concentrations of fibronectin, laminin-111, collagen IV, hyaluronic acid, and perlecan. Those efforts demonstrated that both the chemical environment and adhesion ligand density have major impacts on intestinal stem cell colony formation. Their findings also indicated that the most abundant intestinal tissue ECM proteins have distinct effects on organoid growth, with the highest growth rate seen using laminin and collagen IV, which are abundant in Matrigel.

Understanding the diverse and complex effects that matrix protein composition has on organoid and tissue growth requires precise knowledge of all the proteins that comprise the ECM. The term “*matrisome*” has been adopted [81] to deal with the complexity of matrices, which can comprise over 300 different extracellular proteins, ECM-modifying enzymes, growth factors, and other ECM-associated molecules [82]. The term matrisome is meant to encompass all the proteins present in a matrix rather than just a few major ECM proteins that are typically the only thing described in publications.

Of note, the influence of receptor–ligand interactions is often studied using individual adhesion receptor–ligand pairs. Matrices in vivo on the other hand, consist of many different ligands, each of which can have different effects on adherent cells. Moreover, RGD-containing ligands like fibronectin can engage multiple cell surface receptors, thereby triggering more complex cellular signaling pathways. Hence, precise control over adhesion receptor–ligand binding pairs is important for controlling cellular behavior in vitro. For example, matrices that engage α3β1 and α4β1 integrins allow endothelial cells to form a mature vasculature in vitro [73], while matrices that engage αvβ3 integrin do not. Importantly, biomaterials can be engineered to create ECMs that engage multiple cell-adhesion molecules. The question that arises then is how combinations of two or more adhesion molecule ligands alter cellular behavior. This important question can be addressed using synthetic hydrogels for 3D cultures.

## 4. Cell–Cell and Cell–Matrix Mechanics and Mechanotransduction

Tissue geometry and tissue function are tightly coupled. Tissue growth and the formation of characteristic tissue morphology are both dictated in significant part by mechanical forces at cell–cell and cell–matrix interfaces [83]. Epithelial tissues in organs like kidney, gut, lungs, and mammary glands fold into various 3D shapes (e.g., spherical, tubular, and ellipsoidal) that are essential for proper tissue function. In general, the formation of diverse epithelial shapes is driven by localized active bending and buckling of the cell monolayer and underlying basement membrane [71,84]. The self-organizing capacity of epithelial cells can be exploited to generate unique 3D folded shapes in vitro. With the advent of 3D organoid and spheroid cultures, it is now possible to recapitulate tissue structure–function relationships in vitro [56]. Achieving a particular 3D structure in vitro relies on the self-organizing and regenerative properties of stem cells, as well as on the presence of the 3D biomechanical microenvironment [85]. Studies have shown that stem cell differentiation patterns depend strongly on the stiffness of the 3D ECM [80]. Similarly, external mechanical stresses impact cell and tissue physiology [86]. Therefore, creating a suitable 3D mechanical environment is vital for obtaining physiologically relevant 3D cellular structures.

Organoid formation results from the collective efforts of many cells, where cell divisions, cell fate decisions, differentiation of multiple cell types, and the extrusion of apoptotic cells are all coordinated across space and time to create a structure that replicates that of a tissue [87]. Importantly, cells within these structures exhibit both genotypic and mechanical heterogeneity. Intestinal organoids developed from a single stem cell initially form a spherically symmetric structure with genetically identical cells but with varying levels of the mechanical sensor YAP1 in their nuclei. An early symmetry breaking event is the formation of the first Paneth cell, which creates a niche for the stem cells through the secretion of WNT3A [56]. The spatial organization of stem cells and Paneth cells then undergoes a coordinated crypt–villus structural transformation driven by synergistic osmotic and actomyosin forces, leading to apical constriction at the crypt [88]. During crypt–villus formation, basal tension is higher in villar cells than in crypt cells, while apical constriction is higher in crypt cells than in villar cells. These imbalances drive the folding of the epithelial monolayer to create the characteristic crypt–villus axis seen in the intestine. These events also imply that the remodeling of cell–cell and cell–matrix adhesions must be coordinated during structural transformations.

Spheroids are created using scaffold-free techniques (see fabrication techniques), wherein cell–cell adhesion is the primary driver of cell aggregation. The cells within spheroids are connected to each other through cadherin-based adherens junctions (AJs), tight junctions (TJs), desmosomes, and gap junctions [89,90]. That said, most mechanobiological studies to date have focused on just AJs, as these junctions respond dynamically to mechanical loads so as to maintain junctional integrity [91]. One of the most important physical properties of collective cells aggregates is *surface tension*, which induces cell compaction and volume reduction, resulting in a spherical morphology. Indeed, surface tension also plays a pivotal role in tissue shape transformations [92]. For example, Messal et al. [93] showed that surface tension imbalance and tissue curvature are fundamental determinants of exophytic and endophytic tumors growth in the pancreas. This and other studies [94,95] have highlighted the significance of surface tension in the self-organization and shape transformations of cell collectives. Both active, non-equilibrium, myosin-dependent forces and passive, cell–cell adhesion-based forces contribute to surface tension [96,97]. Because cell aggregates and tissues exhibit both liquid-like and solid-like characteristics, surface tension can exhibit complex oscillatory behaviors [98]. Surface tension can be measured using micropipette aspiration techniques [99] or using compression under parallel plates [100]. For example, Yousafzai et al. [21] used a micropipette aspiration technique to demonstrate myosin force-dependent effects on surface tension in spheroids. Of note, the surface tension of cell aggregates has a size dependence that is unlike liquid droplets, which exhibit a constant, size-independent surface tension. As a result, both pressure and cell density are higher in small aggregates. Yousafzai et al. also showed that a gradient in surface tension can create rapid cellular flows that resemble Marangoni flows [101]. This finding represents a new form of tension-induced collective cellular flow. How Marangoni-like flows emerge in spheroids in the presence of a matrix, as well as the timescales of this phenomenon, require further investigation.

Cell-generated forces can deform, stretch, and stiffen the surrounding matrix. Internal actomyosin-dependent forces that are transmitted to the ECM through integrin-based focal adhesions are known as *traction forces* [102,103]. Most measurements of cell-generated traction forces are carried out using a stiffness-centric approach, where ECM-coated soft substrates are made using polyacrylamide (PAA), polydimethylsiloxane (PDMS), or polyethylene glycol (PEG) that approximate spring-like responses, and where traction forces are then measured by approximating Hookean responses [33,78,104,105]. Cells also sense ECM stiffness and gradients of stiffness through both integrin clustering and conformational changes in mechanosensitive adhesion molecules, which lead to the recruitment of adhesion components like FAK, Talin, Vinculin, Paxillin, and Zyxin. The transduction of mechanical signals to the nucleus generated at cell-ECM boundaries and at cell–cell boundaries is termed *mechanotransduction* [106,107]. Cells respond to mechanotransduction signals based in part on the type of integrin receptor expressed in the cells and its ligand in the ECM. Mechanotransduction can occur through biochemical signaling pathways like Rho-ROCK, Wnt/b-catanin, TGF-B, Ras/MAPK, and P13K/Akt, through physical coupling between the ECM, the cytoskeleton and the nucleus, or by activating mechanosensitive channels. All of these routes are regulated to maintain homeostasis, as mechanical stimuli are often not specific. The mechanical surveillance capabilities of cells that are provided by the molecular machinery underlying mechanosensing allows them to probe ECM stiffness and to discriminate between soft and rigid ECMs [15]. This ability allows cells to sense even minute mechanical gradients and to migrate from soft to rigid areas of the ECM, a phenomenon known as *durotaxis* [108]. Unlike *chemotaxis*, where cells move towards a chemical gradient that they detect using membrane-bound receptors, it remains unclear how cells convert the mechanical information obtained during durotaxis into biochemical responses.

In terms of material properties, *stiffness* has been shown to influence tissue development, differentiation, disease, and regeneration [14,109]. The terms stiffness and elasticity are used interchangeably in mechanobiology. Elasticity corresponds to the equilibrium Young’s modulus, which describes the resistance of the tissue to an applied force when the force is applied extremely slowly (quasi-statically), and is presented as the elastic modulus of the material. An elastic material has a linear stress–strain response that can be computed from the tangent to the stress–strain curve generally in units of Pascal (Pa) or kilo-Pascal (kPa) [N/m]. The elastic moduli of tissues range from 0.1 Pa to 100 kPa, with brain tissue being very soft (~0.1 Pa) and bone being very stiff (~100 kPa). Changes in tissue stiffness can occur in disease conditions and have been used as hallmarks of certain cancers [110]. To recapitulate the same stiffness in 2D, synthetic hydrogels such as PAA and PDMS are used, as they offer a wide range of tunable stiffness [78].

The collective migration of cellular clusters plays an important role in wound healing and collective cancer invasion. To explore these migration mechanisms, the wetting of liquid droplets approach is employed. In this method, PAA substrates of varying stiffnesses are used to allow cellular aggregates to spread in a 2D context. When combined with Traction Force Microscopy, this approach can be used to measure force patterns that occur during collective cell migration [78], the emergence of leader–follower migration modes [111], and active wetting and dewetting [36]. The wetting (spreading) of cell aggregates depends on the stiffness of the substrate [77,112] and the level of cell: substate adhesion [78]. How stiffness sensing is mediated by changes in adhesion levels is not yet clear. Interestingly, when the surface tension stresses of aggregates are comparable to the bulk elasticity of their matrix, a physical property known as electrocapillary interactions arises. These interactions promote wetting of sarcoma cell aggregates on 2D PAA substrates with stiffnesses below 3 kPa. Electrocapillary effects also increase spheroid pressure and drive collective cellular motion. This novel mode of migration differs from the canonical, traction-based mode of cellular migration seen on hard surfaces. An example of pressure-driven cell motion in 3D has been also reported by Raghuraman et al. [113]. They embedded 3D aggregates in soft collagen matrix (0.5 mg mL^−1^) and observed pressure-driven coordinated cellular motions they called cell bursts. Further investigation of the role of electrocapillary interactions and pressure-driven motions in embryonic migration [114], tissue fusion [115], and cancer metastasis [116] will contribute to a deeper understanding of these mechanisms.

**Figure 4:**
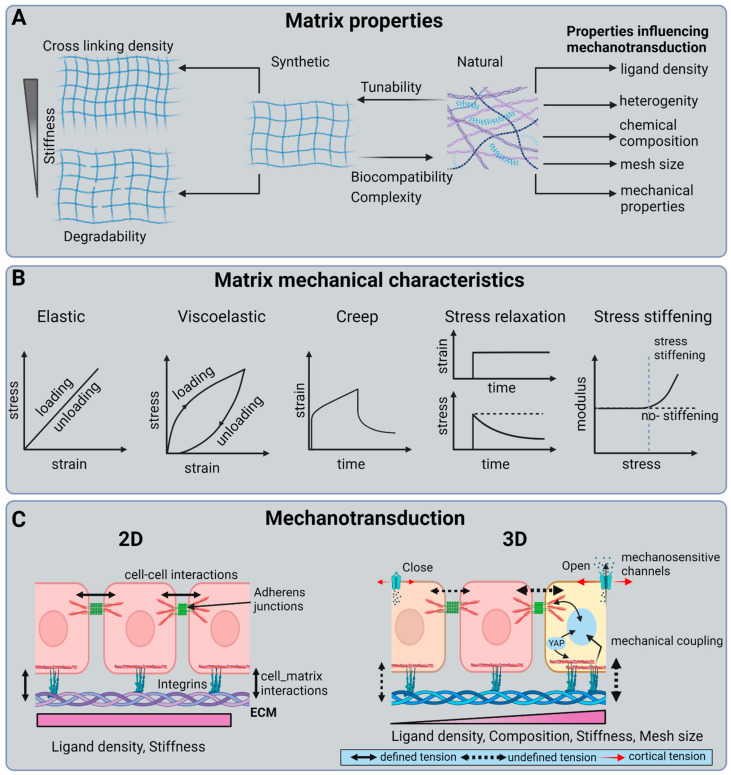
Matrix composition, material properties, and mechanotransduction: (**A**) Matrix properties: Synthetic matrices are simple and allow matrix composition to be tuned to achieve specific mechanical properties. Natural hydrogels, on the other hand, are complex and hard to manipulate. (**B**) Matrix mechanical properties: an elastic material has a linear stress–strain response. That said, most hydrogels are nonlinear, i.e., viscoelasticity and non-linearity increases with increasing chemical complexity. (**C**) Mechanotransduction: mechanotransduction in 3D is more complex than in 2D. Cells in 2D sense uniform ligand density and substrate stiffness. In 3D, on the other hand, there is spatial variation in ligand density, composition, mesh size, and stiffness. Furthermore, cell populations exhibit heterogeneity and, hence, varying mechanical sensitivities.

Another material property that is gaining interest in mechanobiology of organoids and spheroids is viscoelasticity [117]. ECMs in tissues are viscoelastic, meaning they exhibit properties of both elastic solids and viscous liquids. While the role of elasticity in cell mechanotransduction is well studied, the role of viscosity is not [118]. Viscoelastic materials exhibit stress relaxation and creep in response to deformation and applied stress, respectively (Figure 3B). Using breast cancer spheroids, Berae et al. showed that increased ECM viscosity enhances cell migration in 2D, in 2D confined environments, and in cell 3D spheroids [119]. Similarly, Wu et al. demonstrated that hydrogels with a similar elasticity but different stress relaxation behaviors promote migration and fusion of mesenchymal stem cell (MSC) spheroids [120]. Importantly, changes in stress relaxation and the timescale at which cells actively probe the tissue can influence the cell’s perception of different ECM mechanics, potentially changing their behavior. Therefore, having accurate knowledge of the mechanical parameters that cells perceive within living tissues is crucial. Moreover, this knowledge can aid in the design of scaffolds for tissue engineering applications that not only better mimic the mechanical parameters cells perceive in vivo but also replicate the characteristics of the structures responsible for that mechanical response (Figure 4).

## 5. Preparation Methods for Organoids and Spheroids

**Figure 5:**
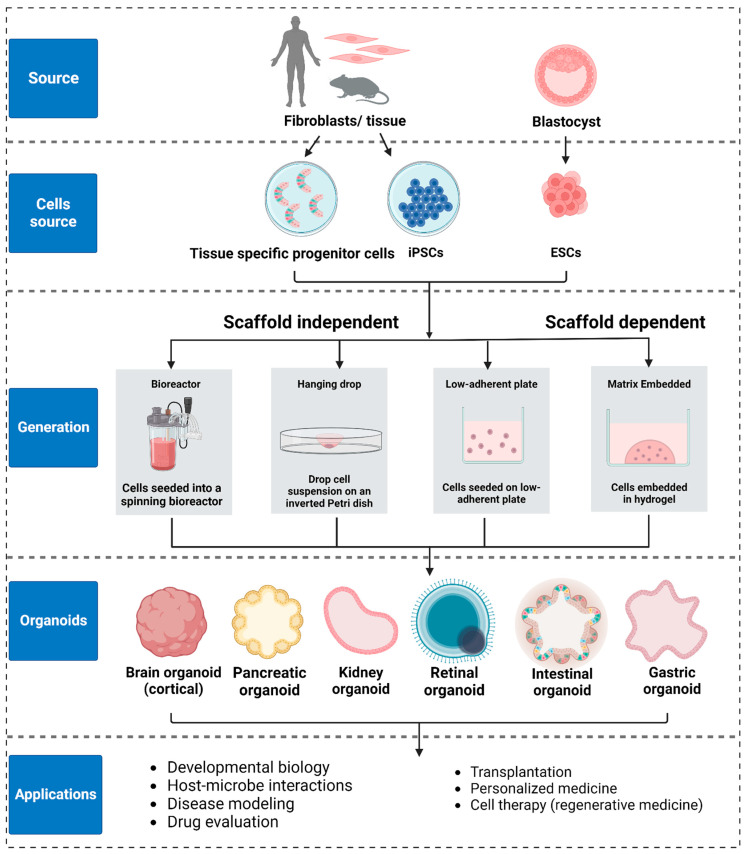
Organoid generation and applications: Organoids can be created using embryonic stem cells (ESCs), induced pluripotent stem cells (iPSCs) and fragments of tissue containing adult stem cells (ASCs). Organoid generation techniques are broadly categorized as scaffold-dependent and scaffold-independent. The choice of generation technique depends on the type of target-tissue model. Organoids are usually generated with scaffold-dependent techniques while spheroids and related tumor models are usually generated using scaffold-independent techniques.

While 2D cultures are widely used, 3D cultures hold greater physiological relevance, making them increasingly popular for both basic and translational research [121]. Organoids and spheroids can be created from multiple cell sources, including embryonic stem cells (ESC), induced pluripotent stem cells (iPSC), adult stem cells (tissue specific progenitor stem cells or tissue fragments) [122], and cell lines. Because organoids and spheroids differ in both structure and basic biology, the choice of cell source and culture protocol depends on the choice of target tissue and the type of study to be carried out (Figure 5). Comprehensive reviews on the choice of stem cells and culture protocols can be found in [85,123,124].

In a landmark study published in 2009, Clever and colleagues showed that an intestinal organoid can be created from a single, Lgr5-positive stem cell. They also showed that intestinal stem cells self-organize into the crypt–villus morphology characteristic of native intestinal tissue, and that they possess all of the cell types found in native intestinal tissue [125]. Subsequently, the development of methods to isolate embryonic stem cells (ESCs) (Thomson et al. [126]) and to generate induced pluripotent stem cells (iPSCs) (Yamanaka group in 2006 [127]) have opened the door to creating 3D cultures from tissue-specific stem cells. For example, Sasai and colleagues reported the first ESC-generated organoid in 2008 [128] and the first iPSC-derived organoid in 2011 [129]. Since then, both ESCs and iPSCs, along with adult stem cells, have been used extensively to create organoid cultures for various tissues including intestine [56,125], stomach [130,131], kidney [132,133], lung [134,135], liver [136,137], pancreas [138,139], breast [140,141], brain [142,143], and optic cup [129,144] (Figure 5). It is important to mention that in the case of ESC- and iPSC-derived models, differentiating them into just one specific single cell type has proven very challenging, and efforts to do this can result in undesirable heterogeneity [17,145]. Recent transcriptomic data on brain and kidney organoids have shown batch-to-batch variabilities, raising concerns about their use in translational applications [146,147]. On the other hand, organoids from adult stem cells obtained from tissue biopsies usually exhibit the relevant pathophysiology, making them suitable for the identification of potential treatments [125].

Three-dimensional culture systems can be classified into scaffold-based and scaffold-free systems. In *scaffold-based systems*, stem cells are embedded in a hydrogel (either natural ones like Matrigel or collagen I, or synthetic ones like polyethylene glycol or alginate). The 3D morphology of intestinal, kidney, and lung organoids created using both culture systems is significantly influenced by cell–matrix and cell–cell interactions [148]. Moreover, both systems can be used for generating 3D structure with low cell–cell adhesion. Scaffold free systems, on the other hand, are only used to generate 3D structures from cells grown in suspension. Cells with high cell–cell adhesion favor the scaffold-free system of growth, and this system is typically used to generate spheroids [149]. Common scaffold-free methods include the spinning method [21], the hanging drop method [150,151], and cell seeding on low adhesion surfaces [152]. The scaffold-free method is a high throughput method, enabling the generation of spheroids of various sizes by controlling cell density (Figure 5).

## 6. Biophysical Tools and Biosensors for Probing Mechanical Properties in 3D

**Figure 6:**
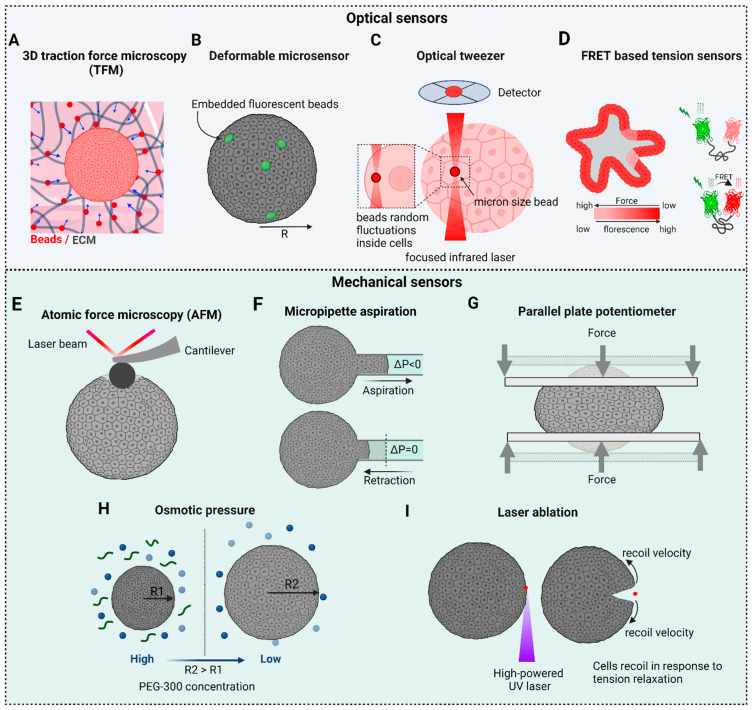
Tools to measure mechanical properties: Optical based sensors: (**A**) Traction force microscopy can be used to obtain 3D force maps occurring during 3D structural rearrangements. (**B**) Deformable microsensors (beads and oil drops) can be used to probe the local mechanical environment within a 3D cell structure. (**C**) Optical tweezers can be used to study intracellular forces and viscoelastic properties. (**D**) FRET-based sensors are used to report spatial and temporal information about forces within a cell. Mechanical based sensors: (**E**) AFM, (**F**) micropipette aspiration, and (**G**) parallel plates are tools used to measure surface tensions and elastic/viscoelastic properties. (**H**) Osmotic perturbations are used to understand pressure-volume regulatory mechanisms. (**I**) Laser ablations can tell us about the elastic and viscoelastic behaviors of cells and 3D tissues.

Our current understanding of how cells generate and perceive mechanical forces stems primarily from the characterization of 2D cultures using a wide variety of biophysical techniques. These techniques, which include atomic force microscopy (AFM) [153,154], micropipette aspiration [99], optical tweezers (OT) [31,41,43], magnetic tweezers (MT) [155], fluorescence resonance energy transfer–based molecular force sensors (FRET) [51,156], and traction force microscopy (TFM) [39,157], have been used to measure the mechanical forces generated by both single cells and cell monolayers. That said, significant technical challenges exist in applying these established biophysical techniques to 3D organoids and spheroids. In this section, we will review the biophysical tools that are being applied successfully to spheroids and organoids (Figure 6).

### 6.1. 3D Traction Force Microscopy (3D TFM)

Tractions forces generated by a cell’s actomyosin cytoskeleton are transmitted to neighboring cells and to the ECM via cell–cell and cell–matrix adhesions, respectively. Traction force microscopy (TFM) provides a powerful tool to measure such traction forces. Single cells and growing organoids apply force on the surrounding matrix by engaging integrin adhesions [33], and the cell-induced deformations of an elastic matrix with known mechanical properties can be then used to quantify traction forces. [158]. Significant limitations include the complexity of the algorithms used to calculate the forces, and the fact that the properties of the 3D ECM are often not fully known. Moreover, TFM measurements made on cell in 3D matrices may underestimate the absolute strain energy as they rely on the assumption of a continuous network, and they neglect the fibrous nature of the network. Additionally, they assume linear elastic material properties and ignore possible strain stiffening responses exhibited by of the matrix [159]. Nevertheless, TFM is a powerful technique that is relatively straightforward to implement and that has contributed significantly to our understanding of cellular mechanosensing and mechanotransduction.

The 3D epithelial organoids emerge in response to collective cell interactions with the matrix. Using 3D TFM, Broguiere et al. showed that the 3D structure of intestinal organoids emerges from differential traction force patterns, and that push-pull forces drive the formation of the crypt–villus morphology [160]. Yousafzai et al. [78] studied collective cell migration by allowing sarcoma cell spheroids to spread on PAA substrates of different stiffnesses (0.7 kPa to 40 kPa). By mapping substate deformations, they showed that traction stresses underneath the spheroids are radiate outward on soft substrates (<4 kPa). Those outward stresses are caused by pressurized cell-flow as spheroids spread on soft substrates.

### 6.2. Deformable Microsensors: Microbeads and Microdroplets

Changes in tissues mechanics are an established hallmark of pathological conditions that emerge from active cellular interactions within a tissue. These interactions give rise to surface properties, represented by surface tension, and bulk mechanical properties, represented by stiffness or bulk tissue elasticity, both of which include ensemble cellular interactions. Tissues in vivo experience both compressive and tensile forces generated by neighboring cells. Compressive stresses are prominent in the case of cancers. Accessing the cell–cell mechanical interactions deep within a tissue, and finding how these mechanical stresses are distributed within the tissue, is difficult due to the inaccessibility of mechanical probes. It is challenging, therefore, to determine how cells interact in a physiologically relevant context, how stresses are distributed among other cells in the tissue, and how these stresses influence cell fate and functionality. Cell-sized, soft, deformable sensors with known elastic properties could be used to measure the mechanical environment of the cells. For example, Dolega et al. [60] used cell-like polyacrylamide microsensors to locally quantify mechanical stress distributions in a living tissue. These sensors had well-defined elasticities, size distributions, and surface coatings that allowed their placement within the tissue. Using this technology, they were able to determine the pressure profile within an organoid by measuring the defamation of the beads. They showed that the anisotropic arrangement of cells within the organoid leads to a pressure rise inside multicellular organoids.

In another interesting study, Donald Ingber and colleagues [62] used fluorescent, adhesion receptor-coated oil microdroplets with defined mechanical properties to measure forces in cell aggregates and living embryonic tissues. Oil droplets were introduced between cells within the tissue, and local anisotropic stresses were determined based on droplet shape deformations visualized by fluorescence microscopy. One drawback of this technique is that while it reports oil droplet shape changes due to anisotropic forces, it cannot measure oil droplet shape changes due to isotropic forces due to the incompressibility of the droplets.

### 6.3. Optical Tweezers

Pioneering work by Ashkin and colleagues laid the foundation for the use of optical trapping and optical tweezers (OT), which have emerged as powerful tools for single-molecule manipulation, nanostructure assembly, cancer cell sorting, targeted drug delivery, and biosensing [42,44,161]. In a typical OT setup, a high numerical aperture (NA) objective (typically 1.2–1.4) is used to form an optical trap by tightly focusing a high-power laser (>1 W), which can trap and manipulate small particles (commonly 0.2 to 5 μm) and apply calibrated forces (<100 pN). OT is actively used for microrheology [162] and for characterizing the mechanobiology of cells in 2D cultures [31,43,104,154,163,164]. While the application of OT to tissues is very challenging, one can use OT in organoids and spheroids to bridge the gap between in vivo and in vitro 2D studies. For example, Guo and colleagues [165] used optical trapping of endocytosed, 0.5 μm diameter particles inside individual cells within a tumor organoid to perform active microrheology. By moving the trapped particles unidirectionally at 0.5 μm/s, they were able to determine a force-displacement relationship that was then used to calculate cytoplasmic stiffness. This effort showed that cells at the center of the tumor organoid are stiff, while cells in the tumor’s outer branches are soft.

### 6.4. Force Biosensors

To characterize the mechanotransduction that occurs across cell–cell and cell–matrix adhesions, one must know the force sensitivity and load-bearing capacity of the adhesion molecules involved, and how they respond to force. Mechanical transduction across these adhesions occurs in pN force range, which can be precisely measured using current biophysical tools [50]. The demonstration that a single myosin motor is capable of generating a force of ~1 pN [166] highlights the potential benefit of identifying the molecular processes that govern mechanosensing and mechanotransduction within these adhesion complexes.

FRET (Förster Resonance Energy Transfer) is a powerful technique to study the mechanobiology of cells and tissues within the context of individual molecules, the proteins ensembles that comprise adhesion sites, and associated cytoskeletal structures [167,168]. FRET-based tension sensors can be genetically expressed within cells to measure molecular scale forces [169]. This technique is based on the transfer of energy from a donor molecule to an acceptor molecule through non-radiative interactions when the two molecules are in close proximity. The energy transfer is distance-dependent, with the transfer only occurring when the distance between the two fluorophores is 1–10 nm [48].

Martin Schwartz and colleagues reported the first calibrated, genetically encoded FRET-based tension sensor for vinculin using the tension sensor module TSMod [50]. Similarly, tension sensor force probes have been developed for other molecules present within adhesion complexes including talin [170], β2-integrin [171], VE-cadherin [172], E-cadherin [173], α-actinin [174], desmoplakin [175] and fibronectin [176]. While most of these studies were performed using cells in 2D, in vivo FRET sensors have been used in mice and flies [177,178]. While FRET is a valuable tool for studying molecular interactions, its application in the mechanobiology of 3D structures is complicated due to issues with spatial resolution, penetration, quantification, background noise, microenvironment variability, and cell heterogeneity.

### 6.5. Atomic Force Microscopy (AFM)

Atomic force microscopy (AFM) is a powerful mechanical indentation technique that has been used widely in biomedical research to measure mechanical properties, surface topographies, and structures of cells and tissues. In its simplest contact mode, the force-distance curve at a single location on the sample can be calculated from the cantilever tip indentation and retraction [153]. Mechanical properties like elasticity, viscosity, and viscoelasticity can be obtained following post-processing using contact mechanics models such as the Hertz model, the Johnson–Kendall–Roberts (JKR) model, the Derjaguin–Muller–Toporov (DMT) model [179], or the Maugis–Dugdale elastic contact model [180]. It is important to mention that in contact mechanics, adhesion between the cell and the probe plays an important role and should be considered when choosing the appropriate model. The Hertz model, however, neglects adhesion between the sample and the indenter.

Depending on the sample shape and size, the cantilever tip can be cylindrical, spherical, pyramidal, conical-shaped, or planar shaped [181,182]. AFM indentation can yield both local and bulk elasticities of organoids and spheroids, as well as viscoelasticity when the cantilever is operated in an oscillating mode. However, AFM cannot give a 3D map of tissue mechanics due it planner application strategy [183].

High-resolution stiffness mapping of tissue mechanics using AFM can be used to identify mechanical heterogeneities in tissues. While matrix stiffening is a hallmark of tumors, malignancy-related softening of tumor epithelial cells is often seen during cancer progression [184]. Importantly, AFM can achieve sufficient resolution to distinguish between individual tissue components (e.g., cells and the ECM) [185]. Cell–matrix mechanical interactions play an important role not only in single cell mechanics but also in spheroid mechanics. For example, AFM measurements by Wang et al. showed that 3D spheroids made of the human adult colorectal adenocarcinoma cell line DLD-1 and grown in a 3D matrix are stiffer than spheroids without matrix supplementation [79]. The apparent Young’s modulus (E, in Pa) was calculated by fitting each recorded force-indentation curve with the Hertz contact mechanics model for a rigid spherical probe.

### 6.6. Micropipette Aspiration

Micropipette aspiration is a versatile and low-cost technique that can be used to measure the viscoelastic properties of cells and tissues. It relies on the aspiration of the cell membrane through a micropipette via the application of negative pressure. Using the Laplace approximation, surface tension and elasticity can be calculated. While it approximates cells and tissues as liquids, it can successfully measure viscoelastic and mechanosensitive behaviors [186].

As discussed above, intestinal organoids undergo complex morphological changes that recapitulate the crypt–villus morphology seen in native intestinal tissue (Figure 2). This asymmetric crypt–villus structure originates from a symmetry breaking event, where a small portion of differentiated cells generate a stem-cell niche through localized, strong actomyosin-dependent *apical constriction (AC)*. AC drives an active shrinkage of the cell’s apical domain, which, in turn, drives epithelial folding. The common players involved in apical constriction are actin, myosin, and Adherens Junctions (AJs) [187]. Using micropipette aspiration to measure the difference in viscoelastic properties at the base of cells occupying the villus and the crypt in intestinal organoids, Yang et al. [88] showed that basal tension in the villus is higher than in the crypt. This spatial differential in apico-basal stress leads to epithelia folding and crypt formation.

Another important physical property of tissues is surface tension, which is essential for determining tissue shape [95], interactions with the surrounding microenvironment [78], and growth dynamics [21]. Surface tension in organoids and spheroids arises from intercellular adhesion and active, myosin-generated contractile forces [94]. Using micropipette aspiration of sarcoma cell aggregates, Guevorkian et al. introduced the measurement of viscoelastic drops through a Creep test (having elastic and viscous response). Using the modified Maxwell model, they deduced the surface tension, viscosity, and elastic modulus of the aggregates. They also observed aggregate mechanosensing as an increase in surface tension upon applied force.

Using aggregates of S180 sarcoma cells with radii ranging from 50 μm to 225 μm, Yousafzai et al. [21] showed that aggregate surface tension is size dependent, with the smaller aggregates having a larger surface tension than larger aggregates. This increase in surface tension was attributed to higher myosin activity in the cells at the outer edge of aggregates [21]. This implies that aggregates of different sizes act as droplets of fluids with different surface tensions. These results contrast with previous results obtained with a parallel plate capacitor to measure surface tension, which showed that cell aggregate surface tension is size independent [188]. Finally, when myosin forces are inhibited using 50 µM Blebbistatin, the size dependence vanishes and the surface tension becomes passive, with only cell–cell junctions contributing.

### 6.7. Parallel Plate Tensiometer

This is a powerful technique to measure the surface tension and viscosity of spheroids using the Laplace equation. [189,190]. In a typical parallel plate tensiometer, two parallel plates rapidly compress a cell mass like a spheroid, causing individual cells to flatten as the mass deforms. At equilibrium configurations, spheroids display a behavior typical of a compressed liquid droplet. Given this, measuring pressure and curvature under compression will yield surface tension. For example, Andolfi et al. used planar AFM macroprobes to measure the viscoelasticity of human oocytes and spheroids [182].

### 6.8. Osmotic Forces

At steady state, the osmotic concentrations of cytoplasmic and extracellular fluids are at equilibrium. Any changes in intracellular or extracellular solute concentrations will generate an osmotic pressure across the plasma membrane that results in the immediate flow of water into or out of the cell until osmotic equilibrium is restored. This equilibration process causes cell swelling or shrinkage, and is known as the volume regulatory mechanism. Cells regulate their volume to alter their mechanical properties in response to their local microenvironment. This regulatory mechanism has been shown to play a role in determining cell function and can even influence stem cell fate. For example, Li et al. used polyethylene glycol (PEG-300) to apply osmotic compression to intestinal organoids. In hypertonic media, organoid volume increased with less crypts and more proliferation though cell volume decreases. The volumetric compression increased intracellular crowding and promoted ISC self-renewal by elevating Wnt/β-catenin signaling. This self-renewal caused the organoid to become more like a cell aggregate.

### 6.9. Laser Ablation

Laser ablation can be used to estimate cellular tensions. Actomyosin contractility generates mechanical stresses in cells and contributes to cortical and membrane tensions that determine cell shape. At the tissue level, these accumulated stresses lead to tissue surface tension, tissue folding and solid stress. In this method, a high-powered laser is used to locally ablate a section of a cell [191], a tissue, or an organoid/spheroid [88,101]. The recoil of surrounding cells or tissue in response to tension relaxation is then monitored. Recoil velocities can be measured using Particle Image Velocimetry (PIV) [101] and the recoil time scales can then be used to estimate the viscoelastic response of the cell, tissue, or organoid. Laser ablation is an invasive technique and, therefore, is not suitable to measure the time evolution of forces. Moreover, measuring stresses quantitatively in 3D using laser ablation is very challenging.

## 7. Biosensors in Organ-on-a-Chip (OoC) Approaches

The self-assembly and regenerative capacity of stem cells embedded within a 3D matrix drive the formation of organ-like structures that are readily accessible to experimental manipulation. That said, one has no control in standard organoid cultures over the structure of the organoids that form, as they emerge and develop stochastically [56]. This is due primarily to the fact that self-emergent structures exhibit stochastic spatial and temporal variations in morphogen and nutrient delivery. While not all functions will vary with variations in organoid shape/structure, some will. Given this, and given that proper organ/tissue functionality requires consistency in the biochemical, nutritional, and biomechanical environment [192,193], investigators have sought out ways to control organoid development. This has been achieved to some extent using the OoC-approach to mimic organ-like microenvironments in vitro [194]. The goal has been to engineer devices in which the constituent elements of the OoC, namely cells and the microenvironment, can be controlled spatially and temporally. More specifically, OoC technology involves the use of various materials to create microfluidic devices that mimic the physiological environments of organs and tissues. The choice of materials depends on factors like biocompatibility, optical transparency, and the ability to replicate the mechanical properties of the target organ. The most common materials used in OoC technology are polydimethylsiloxane (PDMS), polymethyl methacrylate (PMMA), polystyrene, hydrogels, polyurethane, and polyethylene terephthalate (PET) [195]. OoC platforms have been developed for various organs, including brain [196], lung [197,198], heart [199], liver [200], kidney [201], and gut [57]. These platforms have potential applications in drug development and toxicology testing, as well as in basic research [202].

**Figure 7:**
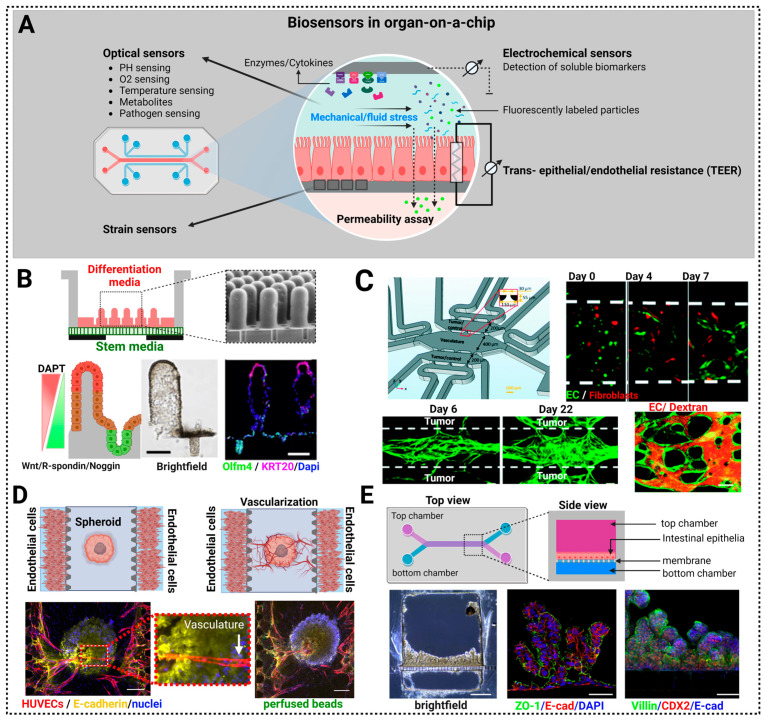
Use of biosensors in organ-on-a-chip approaches: (**A**) Biosensors types used in organ-on-a-chip technology. (**B**) Collagen scaffolds are created to model physiological morphological gradients in vitro through diffusion from the source (lower chamber with stem cell media) to the sink (upper chamber with differentiation medium). A bidirectional gradient of Wnt, R-spondin, Noggin, and DAPT localized the proliferative cells (stem cells: green) to crypts and the absorptive cells (enterocytes: red) to the villus. Immunofluorescence staining for Olfm4 (stem cell marker) and KRT20 (terminally differentiated cell marker) showing tissue polarity under the combined growth factor and DAPT gradients. Scale bar is 100 μm. Adopted with permission from [230]. (**C**) Tumor-on-a-chip platform using endothelial cells (green) in the presence of fibroblasts (red) to create a microvascular network. Confocal slice of a microvasculature (green) that was confirmed through perfusing fluorescently labelled dextran (orange). The vessels from the vasculature chamber extends to the outside chambers to vascularize the tumor organoid. Adopted with permission from [231]. (**D**) Microfluidic platform to recapitulate tumor vasculature. Spheroids are embedded in the central channel and vascularized by the angiogenic sprouts emanating from the endothelial cells seeded in the side channels. The vascular lumens within the spheroids were confirmed through microbead perfusion. Scale bar is 200 µm. Adopted with permission from [222]. (**E**) A micro-engineered approach used to recreate a human intestinal organoid on a chip. The cross-sectional brightfield image of a chip that was exposed to continual media flow at 30 μL/h for 14 days shows complete chip coverage. Scale bar is 250 μm. ZO-1 (green) staining shows 3D epithelial polarization and folded morphology. Staining for E-cadherin/CDH1 (red), and, DNA (blue). CDX2 (red), and Villin (green) demonstrates that this structure is derived from the intestinal brush border. Scale bars is 50 μm. Adopted with permission from [201].

The integration of OoC technology with biosensors is a promising approach that will allow real-time monitoring and analysis of the physiological responses of cells and tissues within microfluidic devices. This integration enhances the functionality and utility of OoC systems for various applications, including drug testing, disease modeling, and toxicity studies [203,204,205] (Figure 7).

**Trans-Epithelial/Endothelial Electrical Resistance (TEER)** is a widely used electrical biosensor for characterizing barrier function and the tightness of epithelial and endothelial tissues and cell layers. TEER has been particularly important in studies of various biological barriers including the blood–brain barrier [206] and the epithelial linings in the gastrointestinal tract [207,208] and lung [197]. TEER provides a measure of the electrical resistance across a monolayer of epithelial cells, as it measures the ability of the cell layer to impede the flow of ions and electrical current. This resistance is due primarily to the presence of tight junctions that exist between adjacent epithelial cells. Tight junctions are protein complexes that create a tight seal around the circumference of adjacent cells to impede the movement of ions and molecules between them. TEER values are typically reported in ohm-cm^2^, so as to account for the surface area of the cell layer being measured [209].

**Electrochemical biosensors** combine the specificity of biological molecules (e.g., enzymes, antibodies, DNA) with the sensitivity of electrochemical techniques to detect and quantify a wide range of biological and chemical substances [210]. These sensors operate on the principle of converting a biochemical reaction or binding event into an electrical signal. A transducer, typically an electrode, converts the biochemical reaction between the recognition element and the analyte into an electrical signal. Common electrode materials include gold, platinum, carbon, and graphene [211].

**Optical biosensors** rely on light properties, such as luminescence, absorption, refractive index, or scattering to detect a biological mechanism. Optical sensors can be used to detect PH, O_2_, H_2_O_2_, and temperature in OoC systems [212,213]. Some excellent reviews on optical biosensors can be found in references [214,215,216].

**FITC-Dextran permeability assays** use fluorescein isothiocyanate (FITC)-labeled dextran to study the integrity of epithelial and endothelial cell barriers, to evaluate drug transport across these barriers, and to assess the effects of various conditions or treatments on barrier function [217,218].

**Strain Sensors** measure and detect mechanical strain or deformation in tissues and cells using strain gauges, piezoelectric sensors, and fiber optic sensors. For example, Lind et al. used a piezo-based strain gauge consisting of flexible cantilevers to distinguish the strain response of cardiac tissues with and without drugs like isradipine and the antidepressant desipramine. Their observations closely matched existing in vivo data, affirming the efficacy of this method for producing dose–response curves via a convenient setup that exhibits a reasonably high throughput [219].

In summary, OoC technology is being used to achieve in vivo-like organ structure and function by providing spatiotemporal control of morphogenetic signaling, nutrient availability (vascularization), and the mechanical environment.

### 7.1. Control of the Morphogenetic Signaling

With regard to controlling morphogenetic signaling, Wang et al. created an intestine-on-a-chip system by micropatterning a collagen scaffold to generate an in vitro, self-renewing, human small intestinal epithelium (Figure 7B). Chemical gradients applied to the crypt–villus axis promoted the creation of a stem/progenitor-cell zone and supported cell migration along the crypt–villus axis. By combining micro-engineered scaffolds and gradients of morphogenetic signals to control the intestinal epithelium in vitro, this exciting study serves as a template to model other tissues that rely on morphogen gradients for differentiation and physiological function. Transwell inserts have also been used to recapitulate the opposing gradients of Wnt and BMP signaling found within the native stem cell niche of the intestinal epithelium. Importantly, these engineered morphogen gradients resulted in in vivo-like tissue compartmentalization wherein stem cells and differentiated epithelial cells were confined to crypts and villi, respectively.

### 7.2. Control of Nutrients for Long Term Growth: Vascularization of Organoids-on-a-Chip

Organoids growing within a 3D matrix receive nutrient supply and oxygen from the growth media via passive diffusion, i.e., with no strict control over the supply. In situations where an even and constant supply of nutrients is essential for reproducible organoid structure and function, a diffusional supply of nutrients and oxygen may not sufficient [212]. This is now being addressed using OoC technology by introducing vascularization to create a constant supply of nutrients and oxygen like seen in vivo [220,221]. By incorporating vasculature, the size and lifespan of organoids can be significantly increased. For example, Shirure et al. [221] used a microfluidic device to drive endothelial cell-dependent vascularization and tumor growth. The device consisted of a central microvascular chamber containing a quiescent, perfused 3D microvascular network and adjacent implantation chambers for examining the impact of chemotherapeutics and anti-angiogenic factors on tumor growth (Figure 7C). This device was capable of maintaining an organoid culture possessing a stable and expanding vascular network for over 20 days. Such vascular networks allow the delivery of nutrients and drugs to tumors, followed by imaging and quantitating tumor growth, cell proliferation, angiogenesis, cell migration, and tumor cell intravasation. In a related study, Nashimoto et al. [222] characterized the dynamic vascularization of co-culture and tri-culture tumor spheroids composed of HUVECs, hLFs, and MCF-7 (Figure 7D). By evaluating tumor activities with or without intraluminal flow over a period of 24 h, they were able to show a significant enhancement of tumor cell proliferation and suppression of tumor cell death following vascularization. Such three-dimensional vascularized models hold great potential as a drug screening platforms by offering an alternative to conventional animal models and two-dimensional cultures.

Finally, it is important to note that vascularization has not yet been demonstrated in pluripotent stem cell (PSC)–derived organoids. One potential hurdle for vascularizing these stem cell-derived organoids is the use of specialized media required for stem cell differentiation, which can potentially interfere with vascular self-assembly and remodeling. Finally, fully functional intravascular perfusion of organoids has only been reported so far through transplantation.

### 7.3. Control of the Mechanical Environment

As highlighted in previous sections (Section 2 and Section 3), the mechanical environment plays a pivotal role in the growth and functionality of organoids and spheroids. Cells in vivo experience mechanical forces that range from single cell-generated traction forces to fluid shear, hydrostatic, and solid stresses arising from neighboring cells and tissues. Because conventional organoids do not provide a means to control mechanical perturbations, they limit efforts to link such perturbations to physiological functions. Organ-on-a-chip technology can now be employed to control these mechanical and hydrodynamic forces in both a spatial and temporal manner.

**Figure 8:**
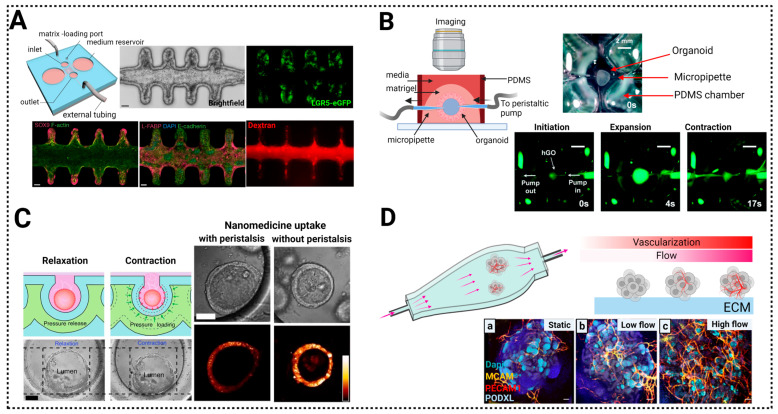
Organ-on-a-chip approaches for organoids and spheroids: (**A**) Scaffold-guided intestinal organoids are grown from LGR 5-GFP-positive stem cells on a tubular patterned-hydrogel. Crypt-like regions exclusively contain progenitor cells (stained for SOX9), while villus-like regions contain enterocytes (stained for L-FABP^+^). Lumen integrity was confirmed by perfusion with FITC labeled dextran (40 kDa). Adopted with permission from [57]. (**B**) Human gastric organoids-on-a-chip are used to model luminal flow in the stomach. Cyclic contraction and expansion cycles were created using a peristatic pump to recapitulate gastric motility. Adopted with permission from [227]. (**C**) Enlarged view of a microwell containing an array of colon organoids subjected to pressure to replicate intestinal peristalsis. Ellipticine-loaded micelles were used to show that peristalsis inhibits the uptake of micelles-encapsulated drug. Scale bar is 50 µm. Adopted with permission from [228]. (**D**) Kidney organoids grown in 3D under shear flow. Shear flow substantially enhances vascularization and maturation of kidney organoids. MCAM (yellow), PECAM1 (red), and PODXL (cyan). Adopted with permission from [229].

One recent example involves the crypt–villus topology of the small intestine [223], where the Lgr5+ stem cells remain localized in the crypt while the nutrient-absorbing enterocytes migrate up to the villus and are eventually extruded into the lumen. The positions of these two cells types within this structure are thought to directed by gradients of Wnt and BMP, respectively [224]. In conjunction with these morphogenetic signals, OoC technology has now demonstrated that a scaffold alone is sufficient to cause small intestinal organoids to assume a crypt–villus topology (Figure 7E). Specifically, Lutolf and colleagues [57] utilized a microchip platform to show that a crypt–villus pattern can emerge on a specialized scaffold that maintained a tubular intestinal epithelia for several weeks, while continuously removing dead cells from the rapidly growing epithelia via perfusion. One of the primary functions of the intestinal epithelia is to maintain barrier function. To evaluate the role of the crypt–villus morphology in the maintenance of barrier function in normal and pathogenic conditions, Workman et al. developed an intestine-on-chip platform that can manipulate the crypt–villus morphology and access the effects of such manipulations on barrier function. In particular, they studied the effects that IFN-β and tumor necrosis factor-α have on barrier function using 4 kDa dextran permeation (Figure 8A) [201].

In addition to mechanical and topological cues, cells and tissue are subjected to changes in hydrostatic pressure to which they must adapt [225]. Hydrostatic pressure is caused by water compartmentalization in cells and tissues, and is particularly important for lumen-forming epithelial tissues and tumors. It is important to note that making microscale pressure measurements in a closed lumen is very challenging. The pressure range in lumens, epithelial domes, and developing mouse embryos, which generally falls between 100 and 300 Pa, can be measured through curvature changes in a fluid/fluid interface as described by Yang et al. [226]. Conventional organoids and spheroids form closed lumens, making them inaccessible to measurements of fluid flow and shear stress. To circumvent this, Lee et al. [227] developed a stomach-on-a-chip system using human gastric organoids derived from human pluripotent stem cells and a bioengineered microfluidic system to introduce luminal flow, thereby modeling in vivo gastric function. They observed peristaltic movements of FITC-labeled dextran coupled to rhythmic cyclic expansions and contractions (Figure 8B). While peristaltic forces are crucial in the digestive tract for maintaining physiological function, they can also promote tumor growth and compromise anti-tumor treatment, as reported by Fang et al. [228]. As depicted in Figure 8C, they grew human colon tumor organoids in microwells and cyclically contracted them using PDMS deformations generated by hydrodynamic pressure. Enhanced expressions of Lgr5 and Ki67 were observed in organoids subjected to peristaltic stresses as compared to those that were not. Their study also showed that organoids exposed to peristalsis exhibit reduced uptake of ellipticine-loaded micelles, suggesting that constant mechanical stimulation reduces drug uptake.

Finally, organoid and spheroid cultures in microfluidic devices are exposed to mechanical stresses from fluid flows. Regulating fluid flow patterns to mimic physiological flows in tissues such as the lungs can be used to characterize the responses of organoids and spheroids to shear stress. Figure 8D highlights the study by Workman et al., who examined kidney organoids cultured on millimeter scale chips and subjected to shear stresses. Their results showed that shear stresses enhanced vascularization and maturation within tubular compartments [229]. In particular, podocytes exhibited enhanced cellular polarity and more adult-like gene expression profiles as compared to the static controls.

## 8. Discussion, Conclusions and Future Directions

Evidence is accumulating from mechanobiology-based studies that the stiffness of the ECM plays a major role in regulating many cellular processes, including gene expression, cell migration, and differentiation [15,109,232]. In conventional 2D cultures, cells are adhered to rigid plastic or glass substrates that have an elastic modulus of ~1 GPa. Most cells in vivo (e.g., embryo, brain, breast, liver, kidney, lungs), on the other hand, are exposed to stiffnesses ranging from 0.1 to 100 kPa [233]. Importantly, the extremely stiff nature of the substrates used for most 2D cultures alter the metabolic and energetic profile of cells, and, as a consequence, their response to many pharmacological perturbations [29,234]. In addition, because conventional 2D cell cultures are typically homogenous, they do not reflect the complexities of cell types found in tissues [5]. Overcoming these limitations will require more robust culture systems that more accurately reflect in vivo conditions.

The 3D culture systems, like organoids and spheroids, have emerged as promising in vitro approaches to mimic the architecture and function of specific organs. They are commonly generated from stem cells grown in 3D matrices that mimic to some extent the microenvironment of the tissue in vivo. Organoids have the potential to revolutionize biomedical research by providing a more accurate and physiologically relevant model for studying development, disease, and drug discovery [8,235]. These 3D structures rely on the self-organizing properties of tissue-specific cells (iPSC, ESC or tissue progenitor cells) and on the availability of 3D biochemical and biomechanical matrices that can support the formation of tissue/organ-like structures [85]. While well-established protocols are available for supporting stem cell growth and subsequent differentiation [235,236,237], the mechanobiological landscapes of these matrices have not been carefully characterized, controlled, or manipulated. Understanding the mechanics of organoids is important for developing accurate and physiologically relevant organoid models, as well as for understanding the behavior of cells and tissues in vivo. Most 3D structures are currently created using 3D matrices like Matrigel or Collagen I, which provide sufficient mechanical signals to support differentiation, and which have provided many insights into the in vivo like behaviors of tissues in health and diseases. That said, Matrigel is derived from a tumor and contains tumor-derived growth factors, potentially limiting its use for growing organoids to be used for drug development and regenerative medicine. Importantly, efforts are being made to design alternative matrices that possess mechanically controllable properties, as these will allow better dissection of mechanotransduction-dependent signaling pathways and growth mechanisms [238]. More efforts are also needed to fully reconstitute the biochemical composition of engineered hydrogels with controllable mechanics that can be tweaked to best mimic native tissue. Better biomechanical probing methods will also yield deeper insights into cellular phenotypes and stem cell differentiation patterns.

Mechanosensing is significantly more complex than chemotaxis, where cells detect chemical signals via surface bound receptors. For example, while ECM stiffness is a bulk material response, how cells really sense the ECM at the micro- and nanoscale level has yet to be explored [232,239]. Similarly, while adhesion ligands like (fibronectin) offer multiple binding sites and specificities [240], how cells can sense and decode mechanical signals to initiate mechanosensitive responses and mechanical homeostasis is far from clear.

Another, major challenge for the field is how to apply in vivo like mechanical, hydrostatic, and hydrodynamic forces to organoids. Cells in tissues are exposed to many environmental forces, including stretching, compression, shear stress from fluid flow, and static mechanical forces. To recreate the microenvironment where organoids are exposed to constant external environmental forces, organ-on-chip platforms can be used. That said, the spatial and temporal regulation of forces has not as yet achieved with these platforms.

Currently, the mechanobiology of tissues and organoids relies on experimental results from studies where ECM parameters such as elasticity, viscoelasticity, ECM degradability, diffusional transport, and dimensionality vary independently. Combining these parameters together increased the complexity of the system.

Mechanobiology combines concepts and techniques from cell biology, physics, mechanics, thermodynamics, materials science, and engineering to explore the complex landscape of the biomechanics of organoids and spheroids. A variety of biophysical techniques can be used to study the mechanical properties of 3D organoids and spheroids, including atomic force microscopy, micropipette aspiration, 3D traction force microscopy, and microfabrication. Importantly, mechanobiology can be merged with synthetic biology to create a new field of “Mechanogenetics” that will enable the harnessing of mechanical signal transduction pathways to control gene expression. The parallel development of the “matrisome” that comprises all ECM molecules involved in embryonic development, organ differentiation, and disease progression will help to precisely identify the signaling cascades that drive growth and differentiation in 3D. Finally, advances in cell-omics technologies, ECM design, imaging, optogenetics, biosensors, and bioengineering techniques will allow us to investigate complex biological processes with high throughput using 3D organoid systems.

In summary, efforts to couple biosensors, which can detect specific biological events, with mechanobiological tools, which can measure intracellular, cellular and multicellular forces, to study organoids/spheroids should provide many new insights into the structure and function of native tissues and organs. Moreover, such efforts should drive the development of novel therapeutic approaches and diagnostics based on mechanobiological principles.

## Data Availability

No new data is generated or analyzed for this review.

## Glossary

- **Biosensor** is a device to detect biological activity or function. - **Optical sensor** is a light assisted measurement of a phenomenon. - **Morphogenesis** is the process by which tissues develop into their final, functional shape. - **Mechanobiology** is defined as the study of the mechanisms by which cells detect and respond to mechanical stimuli. - **Mechanotransduction** is defined as the mechanism through which cells sense, integrate, and convert mechanical stimuli into biochemical signals. - **Mechanosensing** refers to the ability of cells to perceive the mechanical signatures of their environment. - **Mechanogenetics** refers to the convergence of mechanobiology and synthetic biology, to design innovative strategies for harnessing mechanical signal transduction pathways to control gene expression. - **Elasticity** is the ability of a material to resume its normal shape after being stretched or compressed. - **Stiffness** refers to a resistance to deformation. - **Viscoelasticity is** the rate dependence of the resistance of the tissue to an applied force. - **Stress relaxation** is a time-dependent decrease in stress under constant strain. - **Strain hardening** is the strengthening of a material by plastic deformation. - **Organoids** are 3D organ-like structures derived from stem cells. - **Spheroids** are spherical solid structures often used to model tumors. - **Scaffolds** are extracellular matrices (natural or synthetic hydrogels) that support the growth of cell is 3D. - **Durotaxis** refers to the directed migration of cells towards an ECM stiffness gradient. - **Chemotaxis** refers to the directed migration of cells towards an increasing chemical gradient. - **Extracellular matrix** (ECM) is a combination of proteins and molecules that support, and give structure to, cells and tissues in the body. - **Matrisome** is the list of all proteins that constitute the extracellular matrix. - **Solid stresses** emerge in a tissue from the structural components related to growth, and represent the sum of the physical forces exerted during growth.
